# The MDM2–p53 Axis Represents a Therapeutic Vulnerability Unique to Glioma Stem Cells

**DOI:** 10.3390/ijms25073948

**Published:** 2024-04-02

**Authors:** Yurika Nakagawa-Saito, Yuta Mitobe, Keita Togashi, Shuhei Suzuki, Asuka Sugai, Senri Takenouchi, Kazuki Nakamura, Yukihiko Sonoda, Chifumi Kitanaka, Masashi Okada

**Affiliations:** 1 Department of Molecular Cancer Science, School of Medicine, Yamagata University, 2-2-2 Iida-Nishi, Yamagata 990-9585, Japan; 2 Department of Neurosurgery, School of Medicine, Yamagata University, 2-2-2 Iida-Nishi, Yamagata 990-9585, Japan; 3 Department of Ophthalmology and Visual Sciences, School of Medicine, Yamagata University, 2-2-2 Iida-Nishi, Yamagata 990-9585, Japan; 4 Department of Clinical Oncology, School of Medicine, Yamagata University, 2-2-2 Iida-Nishi, Yamagata 990-9585, Japan; 5 Research Institute for Promotion of Medical Sciences, Faculty of Medicine, Yamagata University, 2-2-2 Iida-Nishi, Yamagata 990-9585, Japan

**Keywords:** glioblastoma-initiating cells, p53 activator, wild-type p53, HDM2, brain tumor, chemotherapy

## Abstract

The prevention of tumor recurrence by the successful targeting of glioma stem cells endowed with a tumor-initiating capacity is deemed the key to the long-term survival of glioblastoma patients. Glioma stem cells are characterized by their marked therapeutic resistance; however, recent evidence suggests that they have unique vulnerabilities that may be therapeutically targeted. We investigated MDM2 expression levels in glioma stem cells and their non-stem cell counterparts and the effects of the genetic and pharmacological inhibition of MDM2 on the viability of these cells as well as downstream molecular pathways. The results obtained showed that MDM2 expression was substantially higher in glioma stem cells than in their non-stem cell counterparts and also that the inhibition of MDM2, either genetically or pharmacologically, induced a more pronounced activation of the p53 pathway and apoptotic cell death in the former than in the latter. Specifically, the inhibition of MDM2 caused a p53-dependent increase in the expression of BAX and PUMA and a decrease in the expression of survivin, both of which significantly contributed to the apoptotic death of glioma stem cells. The present study identified the MDM2–p53 axis as a novel therapeutic vulnerability, or an Achilles’ heel, which is unique to glioma stem cells. Our results, which suggest that non-stem, bulk tumor cells are less sensitive to MDM2 inhibitors, may help guide the selection of glioblastoma patients suitable for MDM2 inhibitor therapy.

## 1. Introduction

Glioblastoma, the most common form of malignant brain tumor in adults, ranks among the deadliest of all human cancers [1,2,3]. With the current standard of care, which consists of maximal surgical resection followed by chemoradiotherapy, the median survival of glioblastoma patients is still approximately 1.5 years, with less than 10% of patients surviving longer than five years [1,2,3,4,5]. One of the factors known to contribute to the poor prognosis of this disease is glioma stem cells, cancer stem cells of glioblastoma, which play key roles in recurrence and therapy resistance. Glioma stem cells not only recapitulate pathway alterations described for molecular subtypes of glioblastoma but also display a high degree of plasticity allowing for interconversion between the different molecular subtypes, and as such, they contribute to the tumor heterogeneity characteristic of glioblastoma [6]. Most importantly, glioma stem cells play a pivotal role in initiating and perpetuating tumor growth due to their self-renewal and differentiation capacity to give rise to more differentiated, non-stem cell progeny as well as themselves [7,8]. Therefore, the elimination of glioma stem cells is presumed to be an integral step in achieving long-term survival and, ultimately, a cure for this lethal disease by preventing tumor recurrence [9,10,11]. However, difficulties are associated with identifying cytotoxic agents that eliminate glioma stem cells due to the markedly stronger resistance of glioma stem cells to conventional chemo- and radiotherapies than their differentiated, non-stem cell progeny [6,12]. Intriguingly in this regard, we and others have revealed that glioma stem cells have unique vulnerabilities that render them “more sensitive” to certain drugs than their non-stem cell progeny [13,14,15,16,17,18]. These studies also suggested that the identification of molecules that are differentially expressed in glioma stem cells and non-stem glioma cells represents an effective approach for elucidating the unique vulnerabilities of glioma stem cells. In the present study, we took advantage of this approach and found that MDM2, the major negative regulator of p53 [19,20], was more highly expressed in glioma stem cells than in their non-stem cell counterparts. Further investigations revealed that the MDM2–p53 axis played a critical role in the control of cell survival specifically in glioma stem cells.

## 2. Results

### 2.1. Differential Expression of MDM2 in Glioma Stem Cells and Non-Stem Glioma Cells

**Figure 1:**
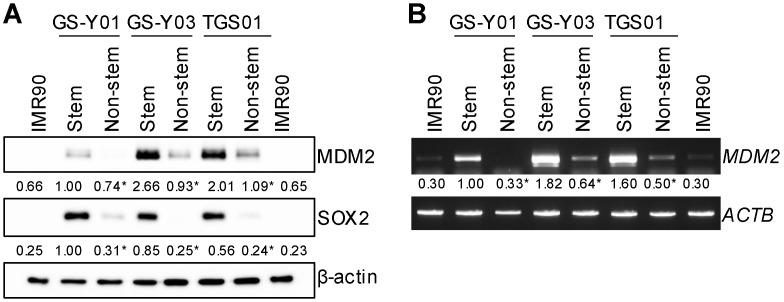
Differential expression of MDM2 in glioma stem cells and non-stem glioma cells. Glioma stem cells and their non-stem cell counterparts (GS-Y01, GS-Y03, and TGS01) as well as human normal fibroblasts (IMR90) were analyzed by Western blot (**A**) and RT-PCR (**B**) analyses for the indicated proteins or mRNAs. The numbers below the Western blot and the RT-PCR images represent the means (n = 2) of the relative band intensities after each band was quantified by densitometry and normalized to the β-actin (Western blot) or the ACTB (RT-PCR) value. * *p* < 0.05 vs. Stem (the same cell line maintained in the stem cell culture condition).

In an attempt to identify molecular vulnerabilities unique to glioma stem cells, we performed an immunoblot analysis of a panel of proteins involved in the regulation of cell survival to detect differential expression in glioma stem cells and their matched/isogenic non-stem cell counterparts. The results obtained revealed that MDM2 was more highly expressed in stem cells (expressing the stem cell marker SOX2) than in non-stem cells (not expressing SOX2) in all three pairs of the glioma stem cell line and its non-stem cell counterpart examined (Figure 1A). Consistent with its protein expression, an RT-PCR analysis showed that the mRNA expression of MDM2 was higher in glioma stem cells than in their non-stem cell counterparts (Figure 1B). Furthermore, MDM2 expression, either at the protein or mRNA level, was negligible in IMR90 normal human fibroblasts (Figure 1A,B). Therefore, MDM2 was more highly expressed in glioma stem cells than in non-stem glioma or normal cells, which prompted us to investigate its role in glioma stem cells.

### 2.2. Glioma Stem Cells Are Dependent on MDM2 Expression to Prevent p53 Activation and Apoptotic Cell Death

**Figure 2:**
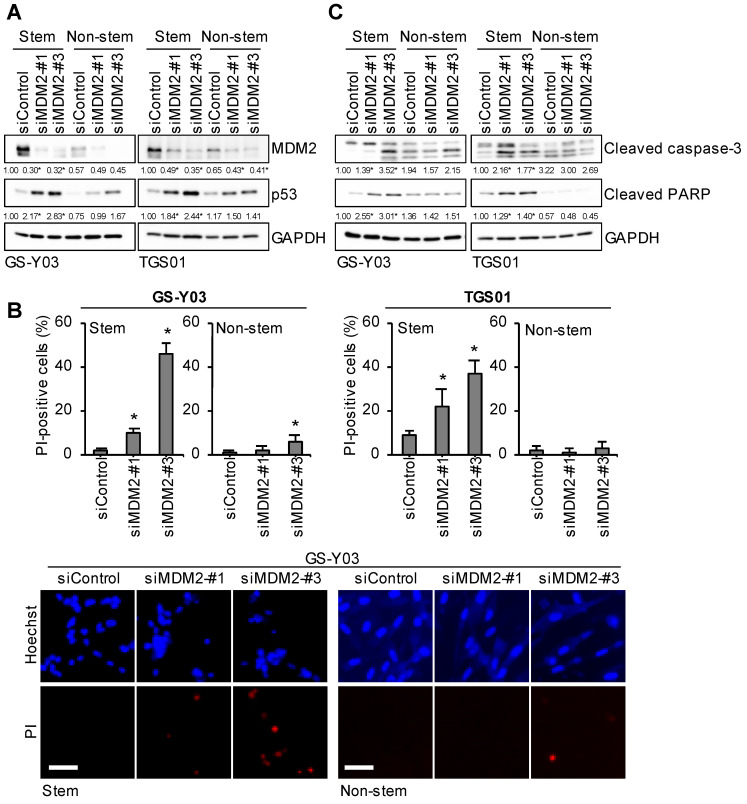
Glioma stem cells are dependent on MDM2 expression to prevent p53 activation and apoptotic cell death. The indicated glioma stem cells and their non-stem counterparts were transiently transfected with an siRNA against MDM2 (siMDM2) or a control RNA (siControl) and cultured for 2 days. (**A**,**C**) Cells were subjected to a Western blot analysis for the indicated proteins. The numbers below the Western blot images represent the means (n = 2) of the relative band intensities after each band was quantified by densitometry and normalized to the GAPDH value. * *p* < 0.05 vs. siControl for each of Stem and Non-stem. (**B**) Cells were subjected to the propidium iodide (PI) incorporation assay to assess the percentage of dead cells (upper panels). Values represent means + SD (n = 3). * *p* < 0.05 vs. siControl. Representative images (GS-Y03) are shown (lower panels). Scale bars: 50 μm.

To establish whether MDM2 plays a critical role in the maintenance of cell survival specifically in glioma stem cells, namely if it is a molecular vulnerability unique to glioma stem cells, we examined the effects of the knockdown of MDM2 on the survival of glioma stem cells and their matched non-stem cell counterparts. When the expression levels of MDM2 were reduced, albeit with different efficiencies, by the introduction of two siRNAs targeting distinct sequences within human MDM2, increases in the expression level of p53 were greater in glioma stem cells than in their non-stem cell counterparts (Figure 2A). Importantly, the knockdown of MDM2 induced more pronounced cell death in glioma stem cells than in their non-stem cell counterparts (Figure 2B). Furthermore, cell death induced by the knockdown of MDM2 was accompanied by the activation of the caspase pathway, as shown by the expression of cleaved caspase-3 and cleaved PARP, which was lower in non-stem glioma cells than in glioma stem cells (Figure 2C). Collectively, these results suggest that glioma stem cells were more dependent on MDM2 expression to inactivate p53 and the apoptotic program for their survival than non-stem glioma cells.

### 2.3. Pharmacological Inhibition of MDM2 Induces p53 Expression and Apoptotic Cell Death Preferentially in Glioma Stem Cells

**Figure 3:**
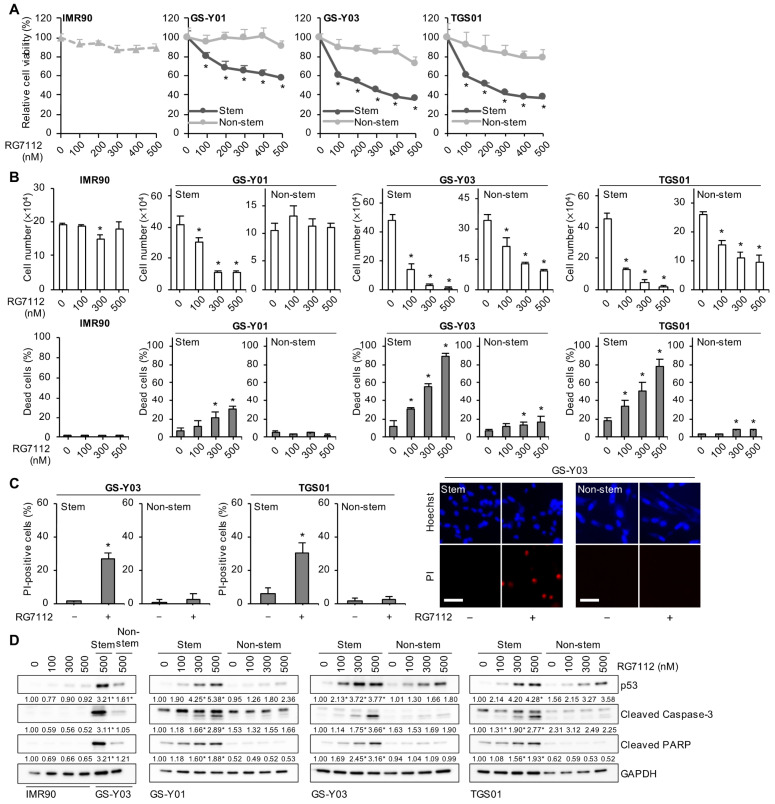
The pharmacological inhibition of MDM2 induces p53 expression and apoptotic cell death preferentially in glioma stem cells. (**A**) The indicated glioma stem cells, their non-stem cell counterparts, and IMR90 cells were treated with RG7112 at the concentrations shown for 3 days and were then subjected to the WST-8 assay. * *p* < 0.05 vs. non-stem cells treated with RG7112 at the same concentration. (**B**) Cells were treated as in (**A**), and the number of viable cells (upper panels) and the percentage of dead cells (lower panels) were then assessed by a trypan blue dye exclusion assay. * *p* < 0.05 vs. cells treated without RG7112. (**C**) Cells were treated without or with 500 nM RG7112 for 1 day. The percentage of dead cells was evaluated using a propidium iodide (PI) incorporation assay (left panels). * *p* < 0.05 vs. cells not treated with RG7112. Representative images (GS-Y03) are shown (right panels). Scale bars: 50 μm. (**D**) Cells treated with the indicated concentrations of RG7112 for 1 day were subjected to Western blot analyses for the indicated proteins. The numbers below the Western blot images represent the means (n = 2) of the relative band intensities after each band was quantified by densitometry and normalized to the GAPDH value. * *p* < 0.05 vs. cells treated without RG7112 (i.e., at 0 nM) for each of Stem and Non-stem. In (**A**–**C**), the values in the graphs represent means + SD (n = 3).

To corroborate the results from MDM2 knockdown experiments, we used a pharmacological inhibitor of the MDM2–p53 interaction, RG7112, the efficacy of which in preclinical models of glioblastoma with its ability to penetrate the blood-brain barrier has been demonstrated [21,22,23]. Consistent with our previous findings [24], RG7112 up to a concentration of 500 nM did not inhibit the viability of IMR90 normal human fibroblasts (Figure 3A). We then treated pairs of the glioma stem cell line and its matched non-stem cell counterpart with different concentrations of RG7112 up to 500 nM and investigated whether RG7112 affected their viability, and the mechanisms by which it did so, in a concentration-dependent manner using the WST assay. The results obtained showed more efficient and concentration-dependent decreases in cell viability after the RG7112 treatment in glioma stem cells than in matched non-stem glioma cells (Figure 3A). Consistent with these results, a cell death analysis using different methods demonstrated that RG7112 induced cell death more efficiently in glioma stem cells than in their non-stem cell counterparts (Figure 3B,C). Furthermore, the RG7112-mediated cleavage of caspase-3 and PARP was proportional to its induction of cell death (Figure 3D). Consistent with these results, RG7112 induced the expression of p53 in a concentration-dependent manner and more efficiently in glioma stem cells than in their non-stem cell counterparts (Figure 3D). These results suggest that the pharmacological inhibition of the MDM2–p53 interaction induced p53 expression and apoptotic cell death preferentially in glioma stem cells over non-stem glioma cells.

### 2.4. MDM2 Inhibition Induces Apoptotic Death in Glioma Stem Cells in a p53-Dependent Manner

**Figure 4:**
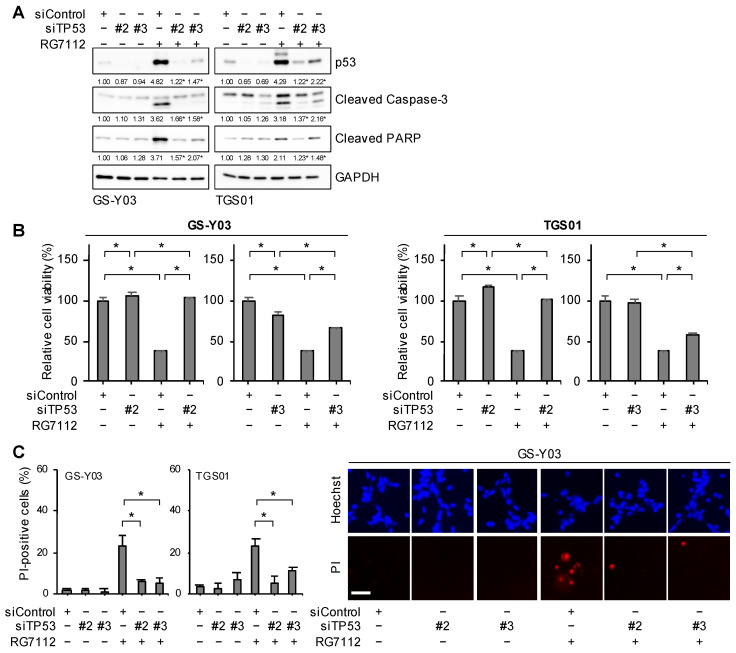
MDM2 inhibition induces apoptotic death in glioma stem cells in a p53-dependent manner. (**A**) The indicated glioma stem cells were transiently transfected with either an siRNA against p53 (siTP53) or a control RNA (siControl). One day after transfection, cells were treated without or with RG7112 (500 nM) for 1 day and then subjected to Western blot analyses for the indicated proteins. The numbers below the Western blot images represent the means (n = 3 for cleaved PARP of TGS01, n = 2 for the others) of the relative band intensities after each band was quantified by densitometry and normalized to the GAPDH value. * *p* < 0.05 vs. cells transfected with siControl and treated with RG7112. (**B**) Cells transfected as in (**A**) were then treated without or with 500 nM RG7112 for 3 days and subjected to a WST-8 assay. (**C**) Cells transfected and treated as in (**A**) were subjected to the propidium iodide (PI) incorporation assay. Representative images (GS-Y03) are shown (right panels). Scale bar: 50 μm. In (**B**,**C**), the values in the graphs represent means + SD (n = 3). * *p* < 0.05.

The present results appear to support MDM2 maintaining the survival of glioma stem cells by inactivating the p53-dependent apoptotic program. To definitively establish the role of p53 in glioma stem cell death induced by the inhibition of MDM2, we examined the effects of RG7112 on glioma stem cells with or without the knockdown of p53. The knockdown of p53 attenuated the reduction in cell viability as well as the induction of cell death and activation of the caspase pathway in RG7112-treated glioma stem cells (Figure 4A–C), suggesting that p53 was required for the apoptotic death of glioma stem cells induced by the inhibition of MDM2.

### 2.5. Increased Expression of BAX and PUMA and Decreased Expression of Survivin upon MDM2 Inhibition in Glioma Stem Cells

**Figure 5:**
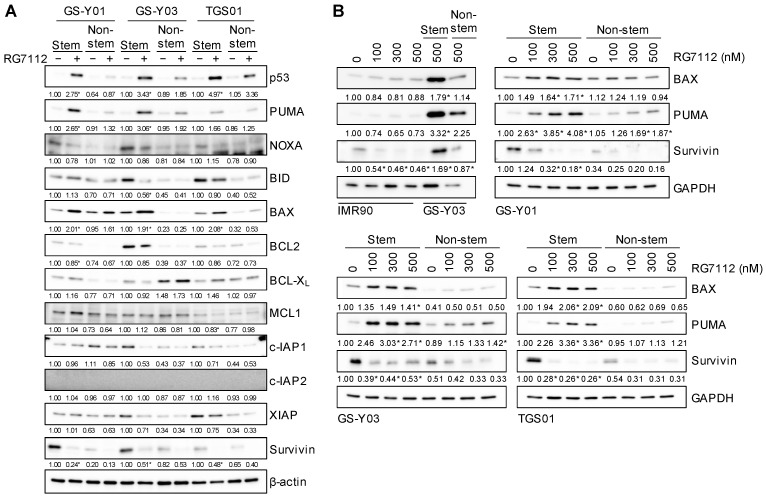
Increased BAX and PUMA expression and decreased survivin expression upon the inhibition of MDM2 in glioma stem cells. The indicated glioma stem cells and their non-stem cell counterparts were treated without or with 500 nM RG7112 (**A**) or with the concentrations of RG7112 shown (**B**) for 1 day and were then subjected to Western blot analyses for the indicated proteins. The numbers below the Western blot images represent the means (n = 2) of the relative band intensities after each band was quantified by densitometry and normalized to the β-actin (**A**) or GAPDH (**B**) value. In (**A**), the band intensity for “Stem” cells treated without RG7112 (i.e., at 0 nM) was set to 1 for each cell line. * *p* < 0.05 vs. cells treated without RG7112 for both Stem and Non-stem, except for the upper left panel in (**B**), where comparisons were made with the left-end lane (i.e., IMR90 treated without RG7112).

We next attempted to elucidate the mechanisms by which the differential activation of p53 by the inhibition of MDM2 in glioma stem cells and non-stem glioma cells resulted in the preferential activation of the apoptotic program in the former over the latter. To this end, we examined the expression of key components of the core apoptotic machinery implicated in cancer cell survival, namely the BCL2 and IAP families, in three pairs of the glioma stem cell line and its matched non-stem cell counterpart treated with RG7112. The majority of the proteins investigated did not show consistent changes in their expression in the three glioma stem cell lines treated with RG7112 or meaningful changes considering their roles in the regulation of apoptosis; however, RG7112 increased the expression of BAX and PUMA, multi-domain and BH3-only pro-apoptotic members of the BCL2 family, respectively [25], and reduced that of survivin (Figure 5A), all of which are known targets of p53 transcriptional activation (BAX [26,27] and PUMA [28,29,30]) and repression (survivin [31,32,33,34]). A detailed analysis of the effects of RG7112 on the expression of BAX, PUMA, and survivin using different concentrations showed that RG7112 increased the expression of BAX and PUMA more in glioma stem cells than in their non-stem cell counterparts (Figure 5B). Furthermore, survivin was expressed at higher levels in glioma stem cells than in their non-stem cell counterparts as previously reported [35], and the RG7112-induced reduction in survivin expression was more pronounced in the former than in the latter (Figure 5B). Collectively, these results suggest that MDM2 maintained the survival of glioma stem cells by inactivating the p53-dependent apoptotic program involving BAX, PUMA, and survivin.

### 2.6. p53-Dependent Changes in BAX, PUMA, and Survivin Expression after the Inhibition of MDM2

**Figure 6:**
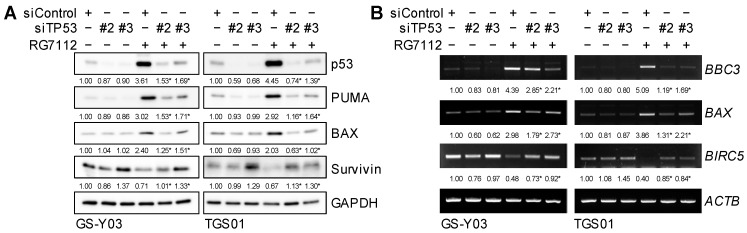
p53-dependent changes in BAX, PUMA (*BBC3*), and survivin (*BIRC5*) expression after the inhibition of MDM2. Cells were transiently transfected with either an siRNA against p53 (siTP53) or a control RNA (siControl). One day after transfection, cells were treated without or with 500 nM RG7112 for 1 day and were then subjected to Western blot (**A**) and RT-PCR (**B**) analyses for the indicated proteins or mRNAs. The numbers below the Western blot and the RT-PCR images represent the means (n = 2) of the relative band intensities after each band was quantified by densitometry and normalized to the GAPDH (Western blot) or the ACTB (RT-PCR) value. * *p* < 0.05 vs. cells transfected with siControl and treated with RG7112.

To confirm whether the inhibition of MDM2 increased the expression of BAX and PUMA and decreased that of survivin through the activation of p53, we examined the effects of the knockdown of p53 on BAX, PUMA, and survivin expression in glioma stem cells treated with RG7112. RG7112-induced changes in BAX, PUMA (*BBC3*), and survivin (*BIRC5*) expression were canceled or attenuated at both the mRNA and protein levels upon the knockdown of p53 (Figure 6A,B), which suggested that BAX, PUMA, and survivin were under the transcriptional control of p53 in glioma stem cells and also that the inhibition of MDM2 modulated the expression of these regulators of apoptosis in a p53-dependent manner.

### 2.7. BAX and PUMA Expression Is Required for the Apoptotic Death of Glioma Stem Cells Induced by the Inhibition of MDM2

**Figure 7:**
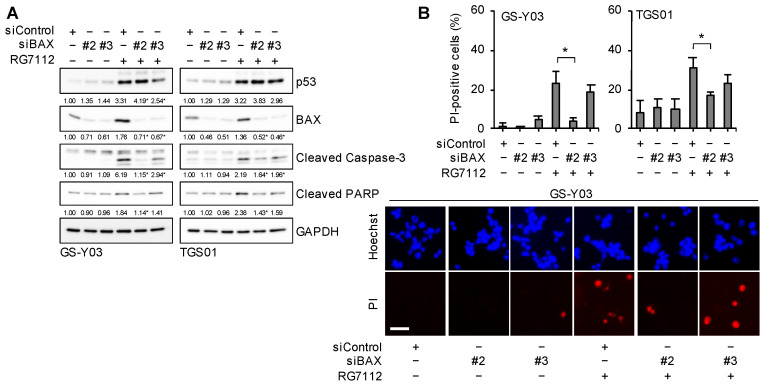
BAX expression is required for the apoptotic death of glioma stem cells induced by the inhibition of MDM2. (**A**) Cells were transiently transfected with either an siRNA against BAX (siBAX) or a control RNA (siControl). One day after transfection, cells were treated without or with 500 nM RG7112 for 1 day and were then subjected to Western blot analyses for the indicated proteins. The numbers below the Western blot images represent the means (n = 2) of the relative band intensities after each band was quantified by densitometry and normalized to the GAPDH value. * *p* < 0.05 vs. cells transfected with siControl and treated with RG7112. (**B**) Cells transfected and treated as in (**A**) were subjected to the propidium iodide (PI) incorporation assay to assess the percentage of dead cells (upper panels). Values represent the means + SD (n = 3). * *p* < 0.05. Representative images (GS-Y03) are shown (lower panels). Scale bar: 50 μm.

**Figure 8:**
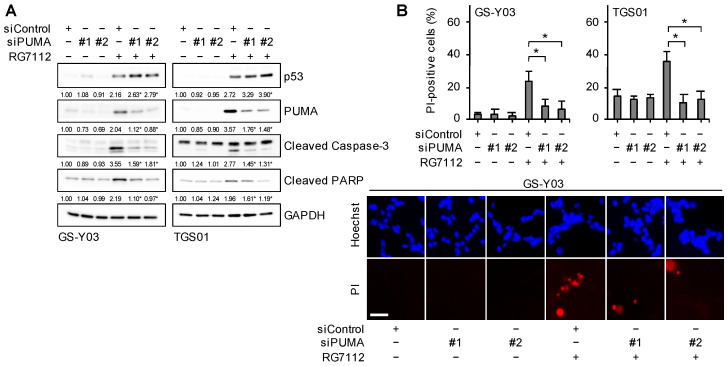
PUMA expression is required for the apoptotic death of glioma stem cells induced by the inhibition of MDM2. (**A**) Cells transiently transfected with either an siRNA against PUMA (siPUMA) or control RNA (siControl) for 1 day were treated without or with 500 nM RG7112 for 1 day. Cells were then subjected to Western blot analyses for the indicated proteins. The numbers below the Western blot images represent the means (n = 2) of the relative band intensities after each band was quantified by densitometry and normalized to the GAPDH value. * *p* < 0.05 vs. cells transfected with siControl and treated with RG7112. (**B**) Cells transfected and treated as in (**A**) were subjected to the propidium iodide (PI) incorporation assay to assess the percentage of dead cells (upper panels). Values represent means + SD (n = 3). * *p* < 0.05. Representative images (GS-Y03) are shown (lower panels). Scale bar: 50 μm.

To establish whether the increased expression of BAX and PUMA plays an essential role in the apoptotic death of glioma stem cells induced by the inhibition of MDM2, we knocked down BAX and PUMA and examined their effects on the apoptotic death of glioma stem cells treated with RG7112. The results obtained indicated that the knockdown of BAX (Figure 7A,B) or PUMA (Figure 8A,B) was sufficient to reduce cell death and inhibit caspase activation induced by the RG7112 treatment, suggesting that BAX and PUMA were both required for the apoptotic death of glioma stem cells induced by the inhibition of MDM2.

### 2.8. Survivin Expression Is Specifically Required for Glioma Stem Cells to Prevent Apoptotic Death

**Figure 9:**
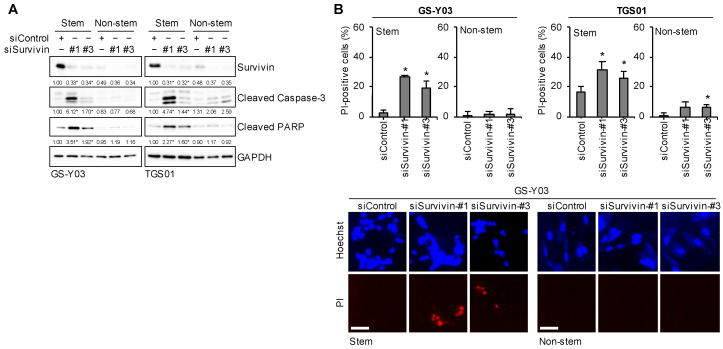
Survivin expression is specifically required for glioma stem cells to prevent apoptotic death. (**A**) The indicated glioma stem cells and their non-stem cell counterparts were transiently transfected with either an siRNA against survivin (siSurvivin) or a control RNA (siControl). Three days after transfection, cells were subjected to Western blot analyses for the indicated proteins. The numbers below the Western blot images represent the means (n = 2) of the relative band intensities after each band was quantified by densitometry and normalized to the GAPDH value. * *p* < 0.05 vs. siControl for both Stem and Non-stem. (**B**) Cells transfected as in (**A**) were subjected to the propidium iodide (PI) incorporation assay to assess the percentage of dead cells (upper panels). Values represent means + SD (n = 3). * *p* < 0.05 vs. siControl. Representative images (GS-Y03) are shown (lower panels). Scale bars: 50 μm.

We previously demonstrated that the endogenous overexpression of survivin was essential for glioma stem cells to maintain their viability under unstimulated conditions [36]. Consistent with the previous report, the present results showed that the knockdown of survivin expression in glioma stem cells led to cell death accompanied by caspase activation (Figure 9A,B). In contrast, the role of the low-level expression of survivin in non-stem glioma cells remains unknown. To test this, we reduced the expression levels of survivin in non-stem glioma cells by introducing the same siRNAs we used for glioma stem cells and found that non-stem glioma cells did not undergo cell death despite reduced levels of survivin expression (Figure 9A,B). Therefore, in contrast to glioma stem cells, non-stem glioma cells may not depend on the expression of survivin for their survival, at least under unstimulated culture conditions. Collectively, these results support the expression of survivin being specifically required for glioma stem cells to prevent spontaneous apoptosis.

### 2.9. The Forced Overexpression of Survivin Protects Glioma Stem Cells from Undergoing Apoptotic Death Induced by the Inhibition of MDM2

**Figure 10:**
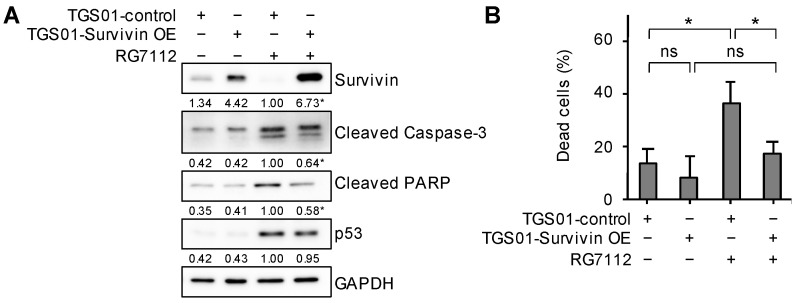
The forced overexpression of survivin protects glioma stem cells from undergoing apoptotic death induced by the inhibition of MDM2. TGS01 cells stably expressing survivin (TGS01-Survivin OE) as well as control TGS01 cells (TGS01-control) were treated without or with 500 nM RG7112 for 1 day and were then subjected to Western blot analyses for the indicated proteins (**A**) or a trypan blue dye exclusion assay to assess the percentage of dead cells (**B**). The values in the graphs represent means + SD (n = 3). * *p* < 0.05. ns, not significant. The numbers below the Western blot images represent the means (n = 2) of the relative band intensities after each band was quantified by densitometry and normalized to the GAPDH value. * *p* < 0.05 vs. TGS01-control treated with RG7112.

Although a reduction in survivin expression was sufficient to induce apoptotic death in glioma stem cells (Figure 9), it was unclear whether this decrease in its expression after the RG7112 treatment promoted the RG7112-mediated apoptotic death of glioma stem cells or if it induced apoptotic death independently of survivin expression levels. To address this question, we established a glioma stem cell subline (TGS01-Survivin OE) that stably overexpressed exogenous survivin as well as a control subline (TGS01-control) (Figure 10A). Under unstimulated culture conditions, TGS01-Survivin OE cells appeared to be less prone to spontaneous cell death than TGS01-control cells (Figure 10B). When these sublines were treated with RG7112, the MDM2 inhibitor induced a significant increase in cell death in TGS01-control cells, but not in TGS01-Survivin OE cells (Figure 10B). Correspondingly, the activation of the caspase pathway upon the RG7112 treatment was weaker in TGS01-Survivin OE cells than in TGS01-control cells (Figure 10A). These results suggest that the reduction in survivin expression played a crucial role in the RG7112-induced apoptotic death of glioma stem cells.

## 3. Discussion

A hallmark of cancer stem cells is their stronger resistance to therapy than their differentiated, non-stem cell progeny, which makes them a challenging target in cancer treatment. Among the factors that contribute to the strong therapeutic resistance of cancer stem cells, such as quiescence and enhanced DNA repair activity, high resistance to cell death, including apoptosis, has been recognized as a key factor [37,38,39]. However, accumulating evidence indicates that cancer stem cells are paradoxically more vulnerable to certain types of stimuli than their non-stem cell counterparts, which supports the idea of cellular mechanisms that comprise vulnerabilities unique to cancer stem cells, namely the Achilles’ heel of cancer stem cells. Previous studies demonstrated that glioma stem cells displayed a unique dependence on oxidative phosphorylation, which was more robust than in non-stem glioma cells [13,14,15,18]. Similarly, the expression of molecules involved in folate metabolism and sensitivity to folate inhibition were higher in glioma stem cells than in non-stem glioma cells [16]. Furthermore, in comparisons with differentiated glioma cells, the de novo pyrimidine synthesis pathway was shown to be up-regulated in glioma stem cells, with rate-limiting pyrimidine synthetic enzymes playing an essential role in the maintenance of glioma stem cells [40]. These findings suggest that unique metabolism in glioma stem cells is a major source of their distinctive vulnerabilities. In the present study, by examining molecules that are differentially expressed in glioma stem cells and their non-stem cell counterparts, we revealed that MDM2, the major negative regulator of p53, was preferentially overexpressed in glioma stem cells. Importantly, the genetic or pharmacological inhibition of MDM2 resulted in the more efficient induction of p53 expression and apoptotic death in glioma stem cells than in their non-stem cell counterparts and normal cells, suggesting that the MDM2–p53 axis represents a hitherto unrecognized, non-metabolic vulnerability of glioma stem cells. The mechanisms underlying the differential activation of p53 upon the inhibition of MDM2 in the absence of external stimuli currently remain unknown; however, the intracellular milieus associated with the stem cell status may generate p53-activating signals that need to be actively counteracted by the overexpression of MDM2 for glioma stem cells to evade p53-dependent apoptosis.

Regarding the downstream pathways of glioma stem cell apoptosis induced by the inhibition of MDM2, the present results showed that, among other pro-apoptotic BCL2 family members known to be transcriptionally activated by p53 [41], BAX and PUMA played a key role in the p53-mediated apoptotic death of glioma stem cells. These results are consistent with previous findings showing the predominant role of PUMA over other pro-apoptotic BCL2 family members in p53-mediated apoptosis [41] as well as the critical role of BAX in PUMA-mediated apoptosis [42,43]. In the setting of the p53-dependent apoptosis of intestinal crypt cells induced by CPT-11, the DNA-damaging chemotherapeutic agent, PUMA was essential for stem, but not transit amplifying cell apoptosis, suggesting a specific role for PUMA in stem cells as a mediator of p53-dependent apoptosis [44]. These findings and the present results indicate that PUMA plays a dominant role over other BH3-only members of the BCL2 family in p53-dependent stem cell apoptosis.

**Figure 11:**
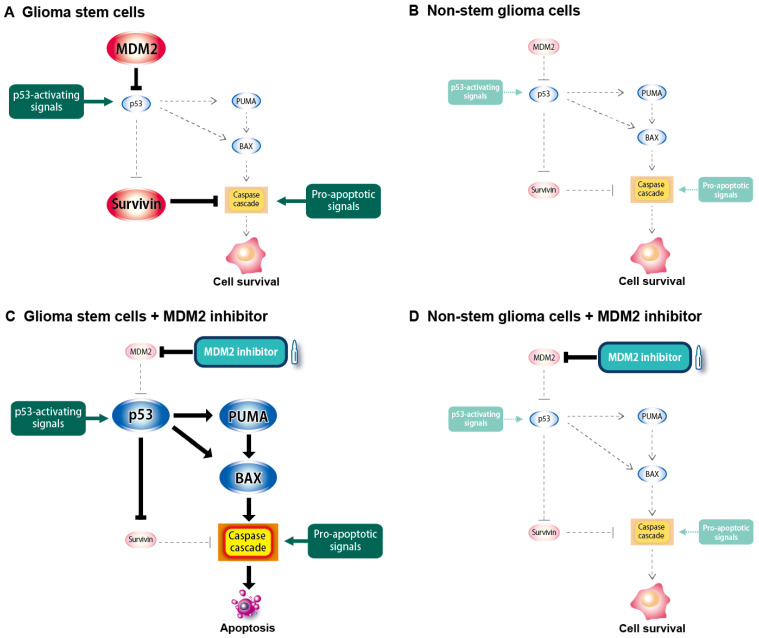
Proposed model for the mechanism underlying the differential role of MDM2 and sensitivity to MDM2 inhibitors between glioma stem cells and non-stem glioma cells. Pro-apoptotic signals associated with the stem cell status, including those activating p53, are generated in glioma stem cells (**A**), but not in non-stem glioma cells (**B**); therefore, the overexpression of MDM2 and survivin is specifically required for glioma stem cells to prevent the activation of the apoptotic program and maintain survival. In glioma stem cells, the inhibition of MDM2 using MDM2 inhibitors causes a p53-dependent increase in the expression of BAX and PUMA and decrease in the expression of survivin, tipping the balance towards apoptosis (**C**). In contrast, the inhibition of MDM2 exerts minimal effects on the viability of non-stem glioma cells, which are not dependent on MDM2 for survival (**D**).

The present results also revealed that survivin was not only a downstream mediator of p53-dependent apoptotic glioma stem cell death induced by the inhibition of MDM2, but was also a unique vulnerability of glioma stem cells, similar to MDM2. Survivin is a member of the IAP family and reportedly inhibits, in conjunction with other members of the IAP family, such as c-IAP1, c-IAP2, and XIAP, the activation of the caspase cascade downstream of mitochondria, where BCL2 family members regulate apoptosis [45,46,47,48]. A previous study showed that survivin was more highly expressed in glioma stem cells than in bulk glioblastoma tissues or non-stem glioma cells [35], which was consistent with the present results. However, it currently remains unclear whether survivin has differential roles in glioma stem cells and non-stem glioma cells. In this regard, the results of survivin knockdown experiments in the present study clearly demonstrated that survivin expression was required for glioma stem cells, but not for non-stem glioma cells to prevent cell death under unstimulated culture conditions, arguing in favor of a unique pro-survival role for survivin in glioma stem cells. Therefore, besides the p53-activating signals discussed above, the stem cell status may also generate apoptotic signals that need to be actively counteracted by the expression of survivin. Together with survivin being under the control of p53 in glioma stem cells, our results suggest that the inhibition of MDM2 is a good approach to eliminating glioma stem cells because it simultaneously targets two layers of their vulnerability. Based on the above discussion, a hypothetical model for the differential role of the MDM2–p53 axis in glioma stem and non-stem glioma cells is presented in Figure 11.

From a clinical point of view, the differential sensitivity of glioma stem cells and non-stem glioma cells revealed in this study has profound implications for how MDM2 inhibitors should be used in the treatment of glioblastoma. In glioblastoma, the p53 gene is mutated in up to 30% of cases [49,50], leaving the remainder with wild-type p53, which makes glioblastoma an attractive target for p53-activating therapy. Previous studies using glioma stem cell lines demonstrated that glioma stem cells were highly sensitive to novel inhibitors of MDM2, such as AMG232 and BI-907828 [51,52], which was consistent with the present study conducted by inhibiting MDM2 both genetically and pharmacologically using RG7112. However, in these studies, the effects of MDM2 inhibitors were not examined using the differentiated or non-stem cell counterparts of glioma stem cell lines [51,52]. In the present study, we investigated the effects of inhibiting MDM2 on the non-stem cell progeny of glioma stem cells for the first time. The results obtained clearly indicated that the loss of the stem cell status was associated with a poor response to the inhibition of MDM2, whether genetic or pharmacological. Notably, RG7112-mediated growth inhibition curves for non-stem glioma cells and IMR90 normal human fibroblasts almost overlapped (Figure 3A), suggesting the lack of or a negligible therapeutic window between normal tissues and the bulk tumor of glioblastoma, which is mostly composed of non-stem tumor cells. In this context, it would be of practical interest and importance to examine whether AMG232 and BI-907828, novel MDM2 inhibitors demonstrated to be highly potent in glioma stem cells, have such a marked differential effect in stem and non-stem glioma cells as RG7112 does. In the meantime, our current results suggest that, in order to fully appreciate their therapeutic potential, MDM2 inhibitors may need to be used in the treatment of glioblastoma patients with a minimal residual tumor burden, whose survival is most likely to be dependent on tumor recurrence initiated by glioma stem cells instead of on the growth of residual tumors driven by the proliferation of non-stem tumor cells. Several clinical trials are underway to evaluate MDM2 inhibitors in patients with glioblastoma [53]. Although the actual sensitivity of stem and non-stem tumor cells to MDM2 inhibitors in patients’ tumors also remains to be investigated, possible differential sensitivity needs to be considered when interpreting the findings of ongoing trials or designing new trials.

In summary, we revealed a crucial role for the MDM2–p53 axis in the control of cell survival that is unique to glioma stem cells. The present results suggest that MDM2 is an Achilles’ heel of glioma stem cells and, thus, has potential as an excellent therapeutic target for patients with glioblastoma. The idea that glioma stem cells and non-stem bulk tumor cells are differentially sensitive to the inhibition of MDM2 may facilitate the selection of glioblastoma patients who will benefit the most from therapies targeting the interaction between MDM2 and wild-type p53. 

## 4. Materials and Methods

### 4.1. Reagents and Antibodies

RG-7112 was purchased from Selleck (Houston, TX, USA) and dissolved in DMSO to prepare a 5 mM stock solution. Propidium iodide (PI) and Hoechst33342 were purchased from Thermo Fisher Scientific (Waltham, MA, USA). Trypan blue solution (0.4%) was obtained from Merck KGaA (Darmstadt, Germany). Antibodies against SOX2 (MAB2018) and MDM2 (AF1244) were purchased from R&D Systems (Minneapolis, MN, USA). An antibody against β-actin (A1978) was purchased from Merck KGaA. Antibodies against GAPDH (#5174), cleaved caspase-3 (#9661), cleaved PARP (#9541), survivin (#2808), Noxa (#14766), BID (#2002), Bax (#5023), c-IAP1 (#7065), c-IAP2 (#3130), XIAP (#2045), and Puma (#12450) were purchased from Cell Signaling Technology, Inc. (Beverly, MA, USA). Antibodies against p53 (sc-126), Bcl-2 (sc-7382), and Mcl-1 (sc-20679) were purchased from Santa Cruz Biotechnologies (Dallas, TX, USA). An antibody against Bcl-XL (10783-1-AP) was purchased from ProteinTech (Rosemont, IL, USA).

### 4.2. Cell Lines, Cell Culture, and Establishment of a Glioma Stem Cell Subline Stably Overexpressing Survivin

The human glioma stem cell lines used in the present study (GS-Y01, GS-Y03, and TGS01) were maintained under previously reported monolayer stem cell culture conditions [16,54]. To obtain the non-stem cell progeny of each glioma stem cell line, the three cell lines were cultured in DMEM/F-12 medium supplemented with 10% fetal bovine serum (FBS) (Thermo Fisher Scientific, Waltham, MA, USA), 100 units/mL of penicillin, and 100 μg/mL of streptomycin for 2 weeks. The non-stem cell progeny was maintained thereafter under the same culture conditions [16]. IMR90, a normal human fetal lung fibroblast line, was purchased from the American Type Culture Collection (Manassas, VA, USA) and maintained in DMEM supplemented with 10% FBS. IMR90 cells with a low passage number (<8) were used in the present study. In all experiments, glioma stem cells were cultured on a collagen I-coated dish or plate, while their non-stem cell progeny as well as IMR90 cells were cultured on a non-coated dish or plate.

To establish a glioma stem cell subline that stably overexpresses survivin, we initially constructed a plasmid that drives the expression of survivin and enhanced green fluorescent protein (eGFP) from a single promoter (pIRES2-Survivin/EGFP) as follows: oligonucleotide adaptor fragments (NEV-Fw: 5′-CGCGGCCGCGATATCG, NEV-Rv: 5′-GATCCGATATCGCGGCCGCGGGCC) were inserted into the *Apa*I/*Bam*HI site of pEGFP-C1 (Takara Bio Inc., Shiga, Japan, GenBank Accession #: U55763) to create pEGFP-C1NEV. Full-length survivin cDNA (*BIRC5* isoform 1, NM_001168.3 from GS-Y03) was amplified by PCR using primers (Survivin-Fw: 5′-TTGTCGACACCATGGGTGCCCCGAC, Survivin-Rv: 5′-TTTTGCGGCCGCTCAATCCATGGCAGCC), and the PCR product was inserted into the SalI/NotI site of pEGFP-C1NEV to create pEGFP-C1NEV-Survivin. The *Sal*I/*Bam*HI fragment containing survivin cDNA from pEGFP-C1NEV-Survivin was then inserted into the *Sal*I/*Bam*HI site of pIRES2-EGFP (Takara Bio Inc., Catalog #6029-1) to construct pIRES2-Survivin/EGFP. The restriction enzymes used in the present study were purchased from New England Biolabs Inc. (Ipswich, MA, USA). These plasmids were fully verified by DNA sequencing.

To establish TGS01 sublines that stably express both survivin and eGFP (TGS01-Survivin OE) or eGFP alone (TGS01-control), TGS01 cells were transfected with pIRES2-Survivin/EGFP or pEGFP-C1 (both carrying the neomycin resistance gene) with LipofectAMINE2000 according to the supplier’s protocol. Transfected cells were cultured in growth medium containing 400 μg/mL G418 over three weeks for the selection of stable pools. Stable transfectants were maintained in the presence of 400 μg/mL G418 thereafter, and stable pools of TGS01-Survivin OE and TGS01-control with similar percentages of eGFP-expressing cells were used in the present study.

### 4.3. Cell Viability/Death Assay

We conducted the WST-8 assay to assess cell viability using Cell Counting Kit-8 (DOJINDO LABORATORIES, Kumamoto, Japan) [15]. The trypan blue dye exclusion assay was performed to count the numbers of live and dead cells as previously described using Trypan Blue solution [15]. The PI incorporation assay was performed to assess the percentage of dead cells as previously described [15].

### 4.4. Western Blot Analysis

A Western blot analysis was conducted as previously described [15]. Cells were harvested and washed with ice-cold phosphate-buffered saline and then lysed in RIPA buffer (10 mM Tris/HCl [pH 7.4], 0.1% sodium dodecyl sulfate (SDS), 1% Nonidet P-40, 0.1% sodium deoxycholate, 150 mM NaCl, 1 mM EDTA, 1.5 mM sodium orthovanadate, 10 mM sodium pyrophosphate, 10 mM sodium fluoride, and protease inhibitor cocktail set III [FUJIFILM Wako Chemicals, Osaka, Japan]). Protein samples whose concentrations were measured using the BCA kit (Thermo Fisher Scientific) were separated by SDS-PAGE, transferred to PVDF membranes, reacted with primary antibodies, and then detected using Immobilon Western Chemiluminescent HRP Substrate (Merck Millipore, Burlington, MA, USA). To reprobe immunoblots, antibodies were stripped from the probed membrane using stripping buffer (2% SDS, 100 mM β-mercaptoethanol, and 62.5 mM Tris-HCl [pH 6.8]). After stripping, the membranes were washed with Tris-buffered saline with Tween 20 and blocked with skim milk. The membranes were then reprobed with appropriate antibodies. Immunoreactive bands were detected by a ChemiDoc Touch device (Bio-Rad Laboratories, Inc., Hercules, CA, USA). Quantification of the bands on the membranes was performed by densitometry using ImageJ software (version 1.53k) (https://imagej.net/ij/, accessed on 19 January 2022).

Original immunoblot images are shown in Supplementary Figures.

### 4.5. Reverse Transcription (RT)-PCR Analysis

An RT-PCR analysis was performed as previously described [24]. Total RNA was extracted from cells using Trizol (Thermo Fisher Scientific), and 1 μg of total RNA was reverse transcribed using the PrimeScript RT reagent kit (Takara Bio Inc.) according to the manufacturer’s protocol. The target genes were amplified with Quick Taq HS DyeMix (Toyobo Co., Ltd., Osaka, Japan) using the primer sets listed below (Table 1). Quantification of the bands in the gels was performed by densitometry using ImageJ software. 

### 4.6. Gene Silencing by siRNA

siRNA against p53 (*TP53*, #2: HSS186390 and #3: HSS186391), MDM2 (#1: HSS142909 and #3: HSS142911), BAX (#2: HSS141355 and #3: HSS141356), PUMA (*BBC3*, #1: HSS146893 and #2: HSS146895), Survivin (*BIRC5*, #1: HSS 179403 and #3: HSS 179405) (Thermo Fisher Scientific), or AllStars Negative Control siRNA (QIAGEN, Venlo, The Netherlands) was transfected using Lipofectamine RNAiMAX (Thermo Fisher Scientific) in accordance with the manufacturer’s instructions.

### 4.7. Data Reproducibility and Statistical Analysis

Western blotting, RT-PCR analyses, and cell viability/death assays were repeated at least twice with similar results, and one set of representative data is presented. Data analyses were performed using the software Microsoft Excel (Version 2402). The significance of differences was assessed using the Student’s two-tailed *t*-test for comparisons of two groups. *p* values < 0.05 were considered to be significant. 

## Figures and Tables

**Table 1:** PCR primer sequences.

Gene Name	Forward	Reverse
*MDM2*	GGTGCTGTAACCACCTCACA	TGAGTCCGATGATTCCTGCTG
*BBC3/PUMA*	TACGAGCGGCGGAGACAAG	AGCACAACAGCCTTTCCTGA
*BAX*	GCTTCAGGGTTTCATCCAGGATCGAG	TGCACAGGGCCTTGAGCACCAGTTTG
*BIRC5/Survivin*	CCTTTCTCAAGGACCACCGCATC	CGTCATCTGGCTCCCAGCCTT
*ACTB*	CCCATGCCATCCTGCGTCTG	CGTCATACTCCTGCTTGCTG

## Data Availability

All data are contained in this article and there are no repository data.

## Supplementary Materials

The following supporting information can be downloaded at: https://www.mdpi.com/article/10.3390/ijms25073948/s1.
