# Supplementary material for: The MDM2–p53 Axis Represents a Therapeutic Vulnerability Unique to Glioma Stem Cells
Source: Int J Mol Sci. 2024 Apr 2;25(7):3948. doi: 10.3390/ijms25073948 (PMC11011437; doi:10.3390/ijms25073948)

Original Western Blot images

Figure 1

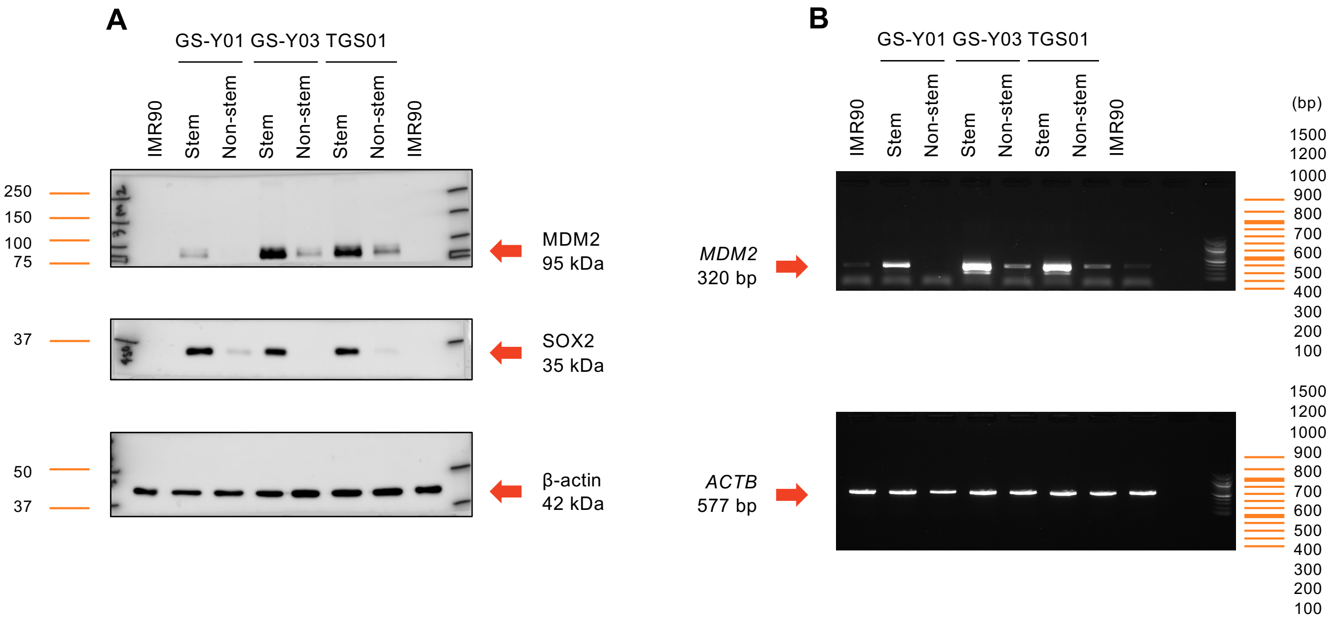

Figure 2

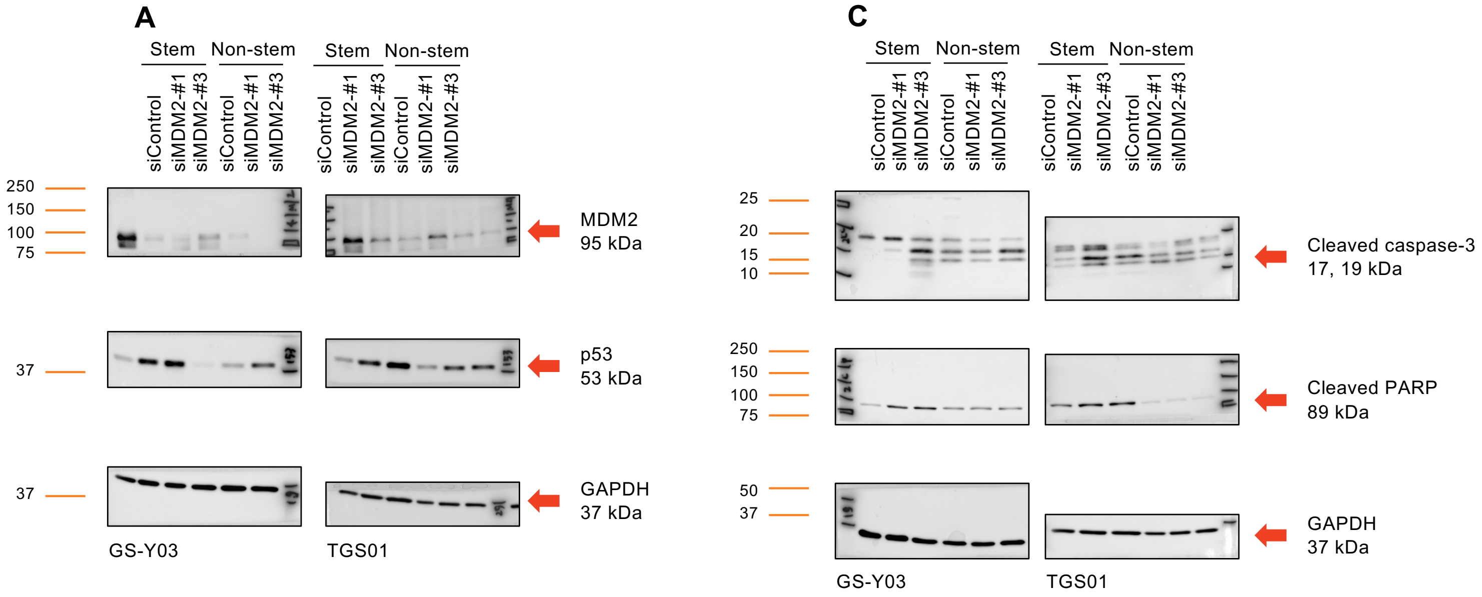

Figure 3

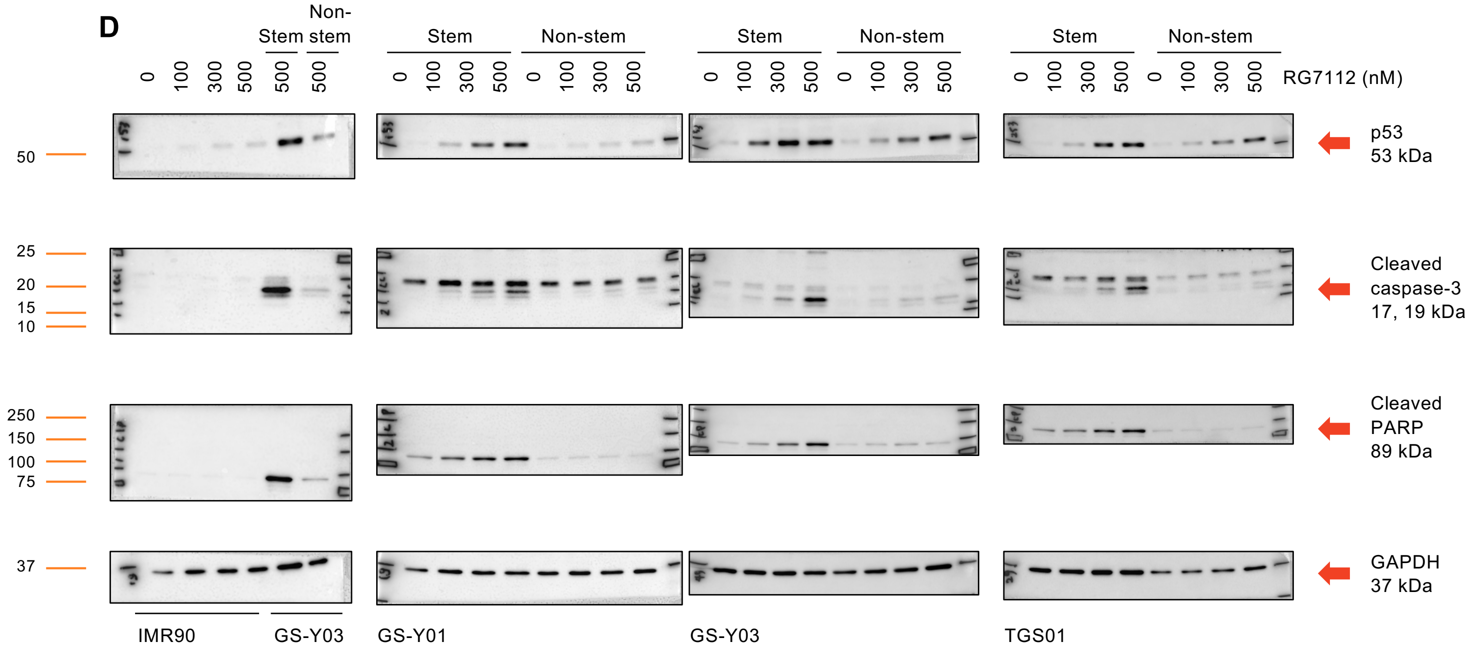

Figure 4

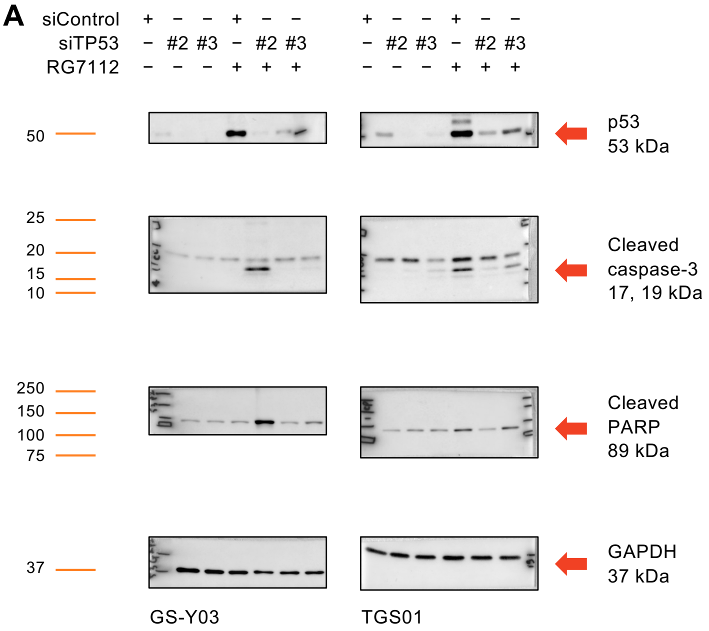

Figure 5

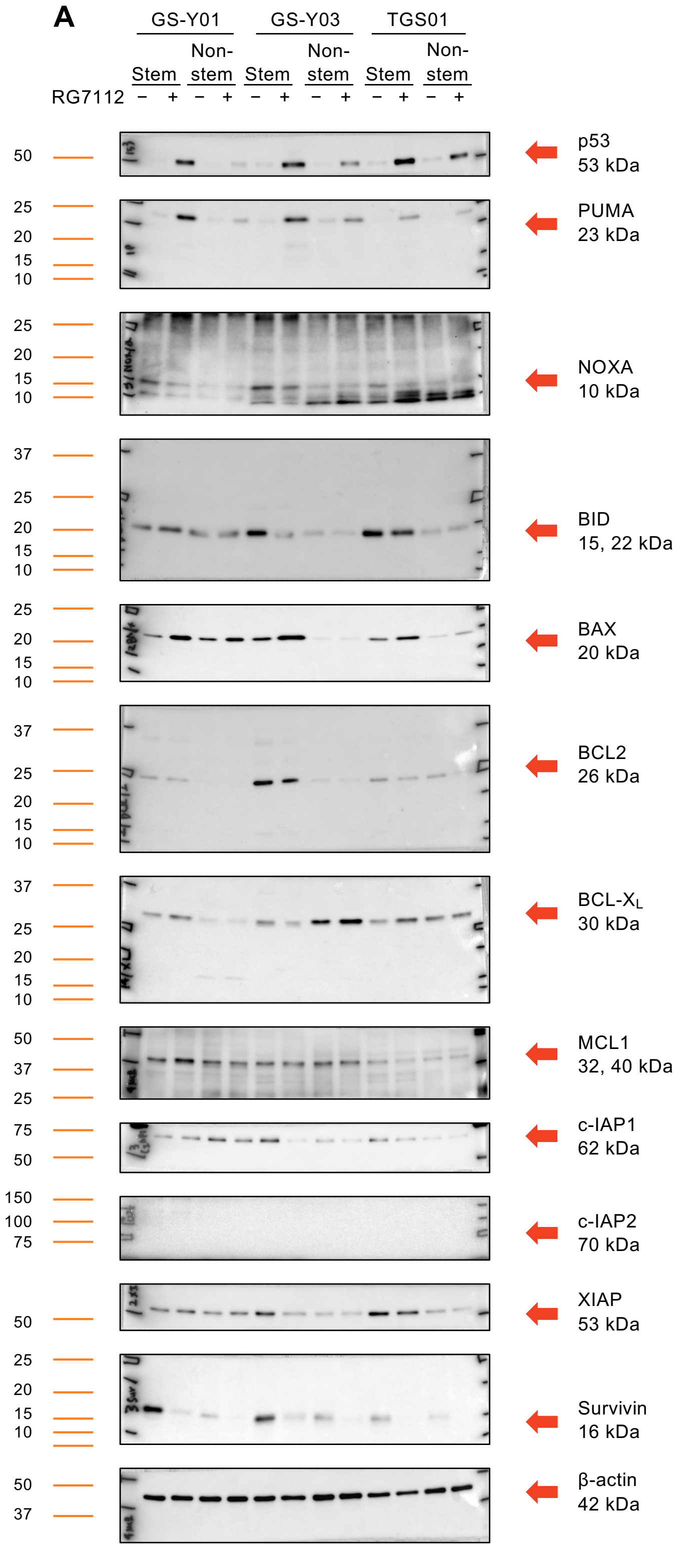
A
GS-Y01
GS-Y03
TGS01
Stem
Non-stem
Stem
Non-stem
Stem
Non-stem
RG7112 − + − + − + − + − + − +
p53
53 kDa
PUMA
23 kDa
NOXA
10 kDa
BID
15, 22 kDa
BAX
20 kDa
BCL2
26 kDa
BCL-X$_L$
30 kDa
MCL1
32, 40 kDa
c-IAP1
62 kDa
c-IAP2
70 kDa
XIAP
53 kDa
Survivin
16 kDa
β-actin
42 kDa
50
25
20
15
10
37
75
150
100

Figure 5

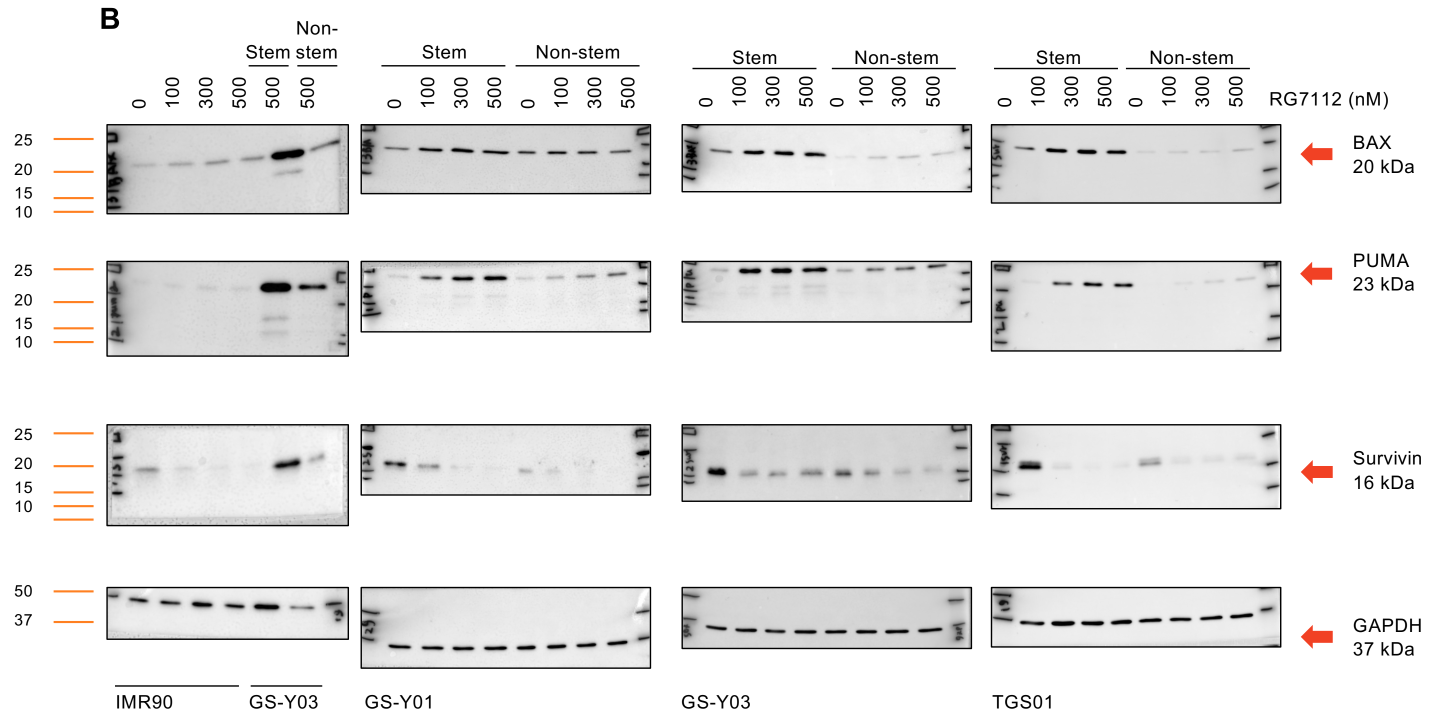

B
Non-
Stem stem
Stem
Non-stem
Stem
Non-stem
Stem
Non-stem
0 100 300 500 500 500
0 100 300 500 0 100 300 500
0 100 300 500 0 100 300 500
0 100 300 500 0 100 300 500
RG7112 (nM)
25
20
15
10
BAX
20 kDa
25
20
15
10
PUMA
23 kDa
25
20
15
10
Survivin
16 kDa
50
37
GAPDH
37 kDa
IMR90
GS-Y03
GS-Y01
GS-Y03
TGS01

Figure 6

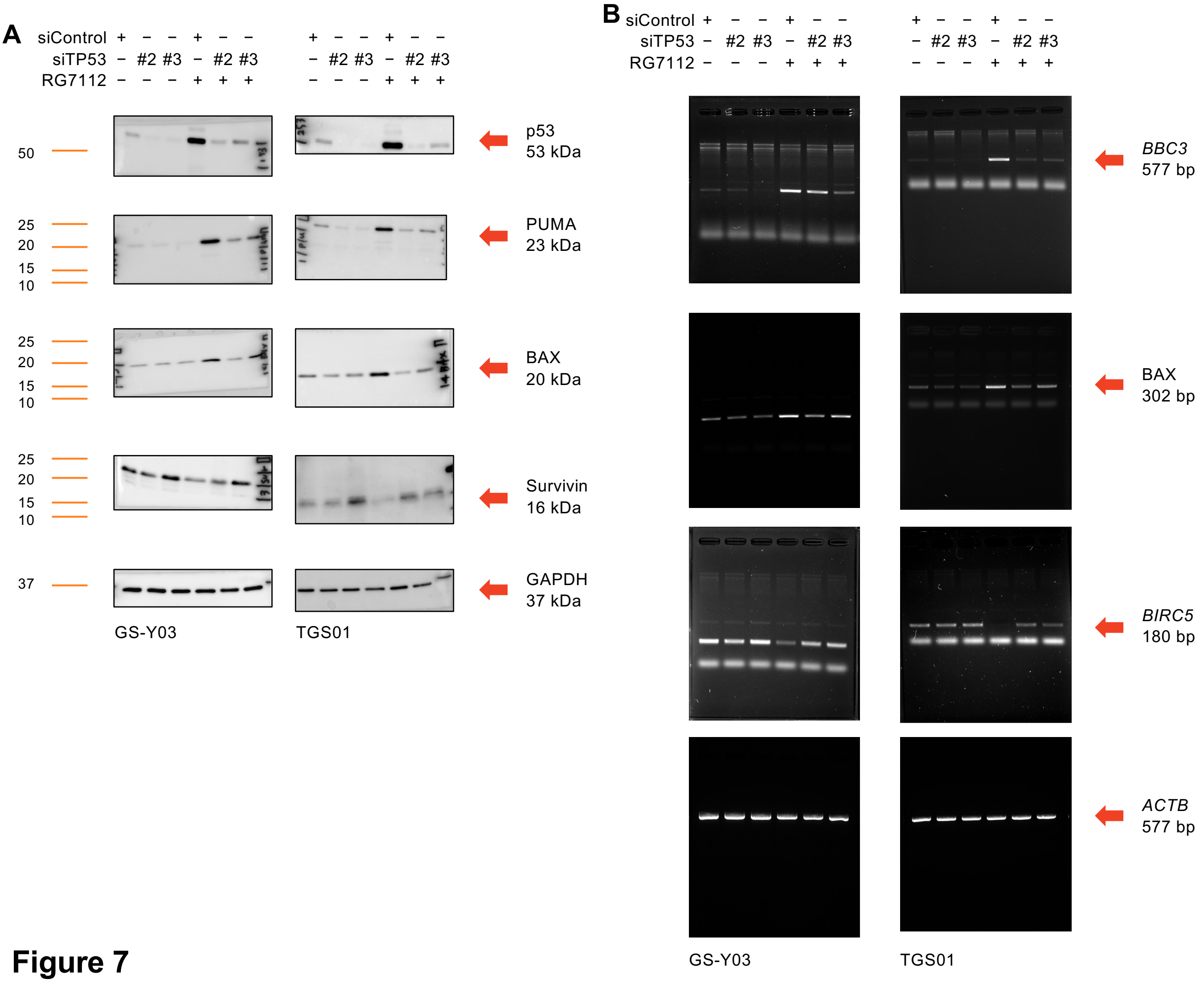

Figure 7

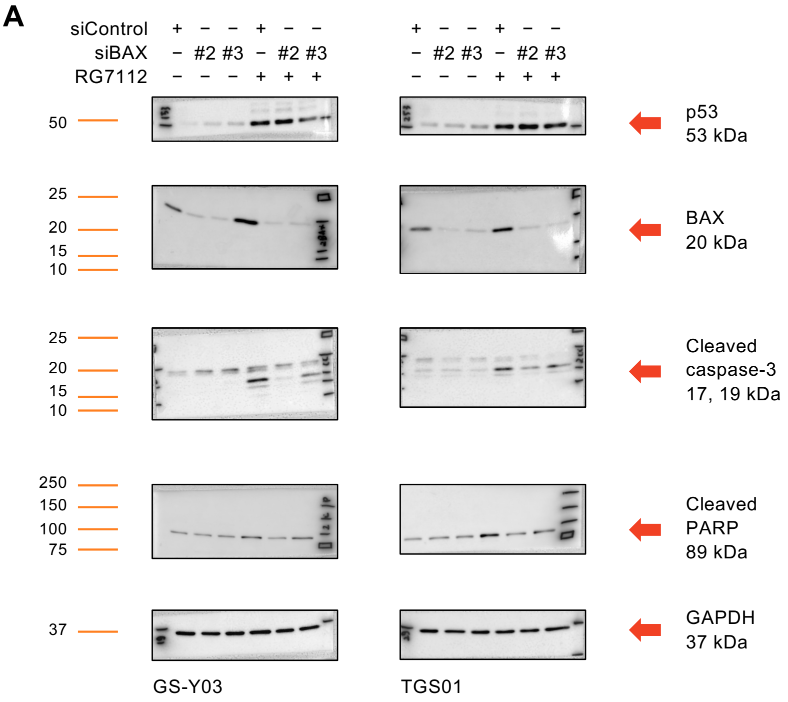

Figure 8

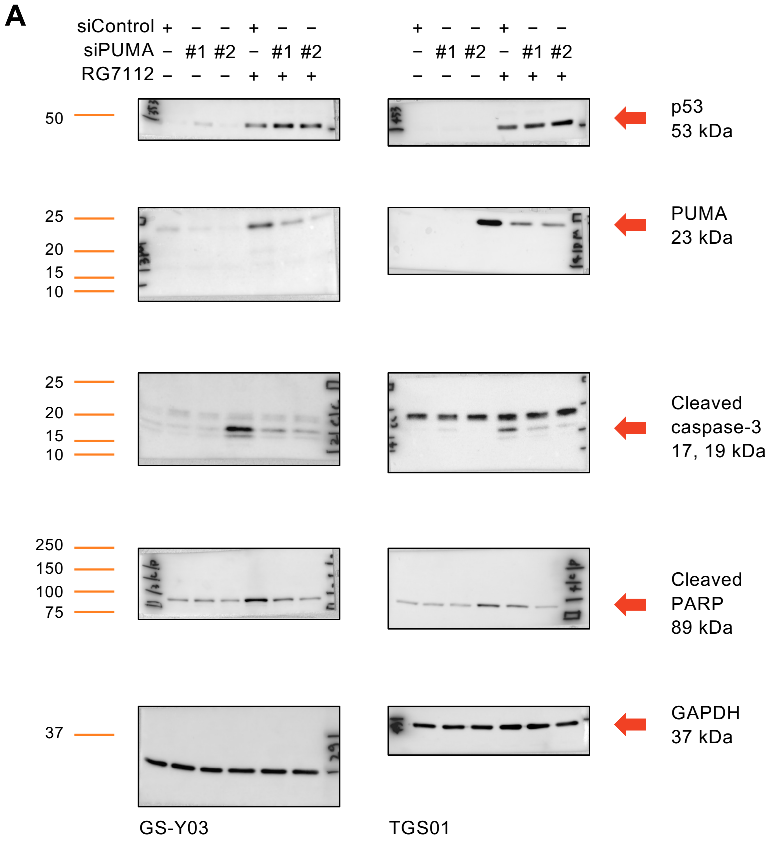

Figure 9

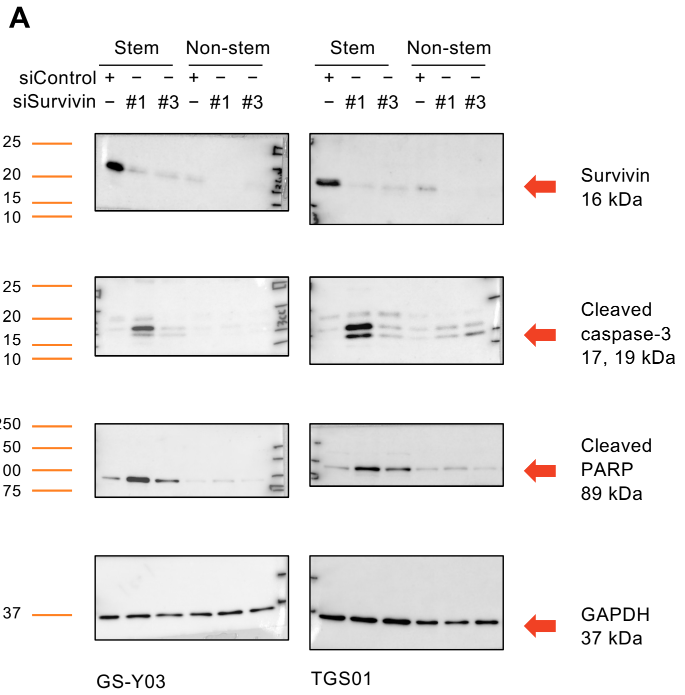

Figure 10

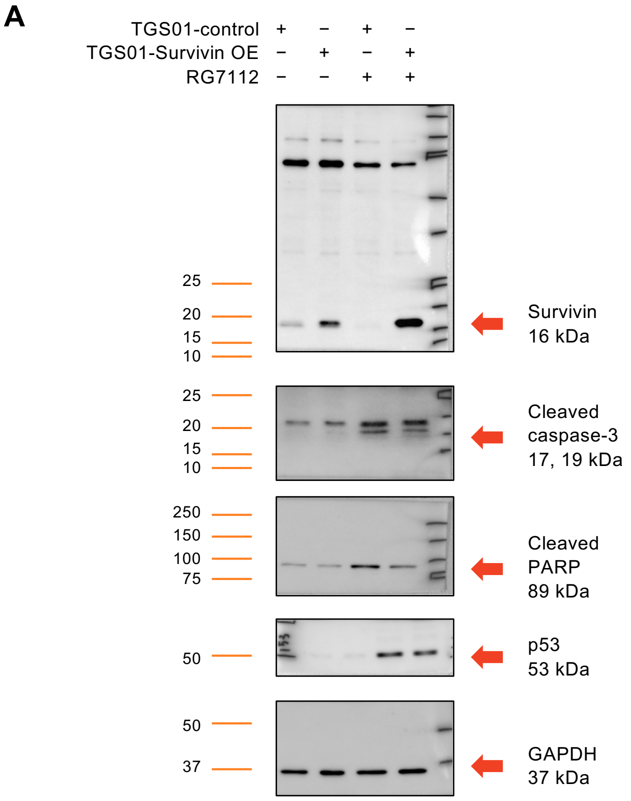

Supplement: Supplementary file 1 [file ijms-25-03948-s001.zip › ijms-2901305-supplementary.pdf]
